# Association of Inflammation-Based Ratios with Endothelial Dysfunction Markers and Clinical Parameters in Limited Cutaneous Systemic Sclerosis

**DOI:** 10.3390/jcm14248806

**Published:** 2025-12-12

**Authors:** Leyla Schweiger, Andreas Meinitzer, Heimo Strohmaier, Florentine Moazedi-Fürst, Viktoria Nemecz, Katharina Kurzmann-Gütl, Marianne Brodmann, Franz Hafner, Philipp Jud

**Affiliations:** 1 Division of Angiology, Department of Internal Medicine, Medical University of Graz, 8010 Graz, Austria; viktoria.muster@medunigraz.at (V.N.); katharina.guetl@medunigraz.at (K.K.-G.); marianne.brodmann@medunigraz.at (M.B.); ordination@graz-internist.at (F.H.); philipp.jud@medunigraz.at (P.J.); 2 Institute of Medical and Chemical Laboratory Diagnostics, Medical University of Graz, 8010 Graz, Austria; andreas.meinitzer@medunigraz.at; 3 Center of Medical Research (ZMF), Medical University of Graz, 8010 Graz, Austria; heimo.strohmaier@medunigraz.at; 4 Division of Rheumatology, Department of Internal Medicine, Medical University of Graz, 8010 Graz, Austria; florentine.fuerst@medunigraz.at

**Keywords:** limited cutaneous systemic sclerosis, inflammation-based ratios, endothelial dysfunction, pulmonary arterial hypertension

## Abstract

**Background**: Limited cutaneous systemic sclerosis (lcSSc) is an autoimmune disease with a wide range of different biomarkers, while inflammation-based ratios have been less extensively investigated. This study aimed to evaluate the associations between inflammation-based ratios, disease-specific parameters, and endothelial dysfunction, as well as to assess the predictive role of inflammation-based ratios in lcSSc. **Methods**: A total of 38 lcSSc patients and 38 matched controls with primary Raynaud’s phenomenon were analyzed at baseline regarding inflammation-based ratios, lcSSc-specific parameters, and parameters of endothelial dysfunction. LcSSc patients were prospectively observed during a 3-year follow-up period in which lcSSc complications were recorded annually. **Results**: LcSSc patients had a significantly higher neutrophil-to-lymphocyte ratio, monocyte-to-lymphocyte ratio (MLR), fibrinogen-to-albumin ratio, monocyte/high-density lipoprotein (HDL) ratio, and neutrophil/HDL ratio versus controls (all *p* < 0.05). During follow-up, the MLR, C-reactive protein (CRP)/albumin ratio, monocyte/HDL ratio, and neutrophil/HDL ratio increased significantly (all *p* < 0.05) in lcSSc patients. The monocyte/HDL ratio correlated positively with the DETECT score step 2 (r = 0.453, *p* = 0.032) and negatively with the UCLA SCTC GIT total score (r = −0.469, *p* = 0.024). The CRP/albumin ratio correlated significantly with the EUSTAR index (r = 0.473, *p* = 0.024) and the fibrinogen-to-albumin ratio correlated with asymmetric dimethylarginine (r = 0.452, *p* = 0.044). The MLR and CRP/albumin ratio were associated with development of pulmonary arterial hypertension (*p* = 0.036, *p* = 0.006), and the lymphocyte/HDL ratio was associated with newly developed interstitial lung disease (*p *= 0.004). **Conclusions**: Readily available inflammation-based ratios may reflect vascular and inflammatory activity and could contribute to risk stratification for pulmonary complications in lcSSc; however, these exploratory findings require confirmation in larger cohorts.

## 1. Introduction

Systemic sclerosis (SSc) is an autoimmune-mediated connective tissue disease characterized by endothelial dysfunction, autoantibody production, and fibroblast dysfunction [1]. It can be classified into three subtypes according to skin involvement: diffuse cutaneous systemic sclerosis (dcSSc), limited cutaneous systemic sclerosis (lcSSc), and SSc without skin involvement. LcSSc is characterized by skin involvement limited to the face and limbs distal to the elbows or knees, with less severe organ involvement compared to dcSSc [2,3]. Although SSc is a rare disease, it has a high mortality rate due to potential organ involvement, including interstitial lung disease (ILD), pulmonary arterial hypertension (PAH), and renal crisis [4,5,6].

Several parameters have been investigated for their association with SSc complications. In particular, inflammatory parameters such as C-reactive protein (CRP), the erythrocyte sedimentation rate (ESR), and ferritin are known to be associated with disease complications in patients with SSc. These markers have been linked to poorer prognosis and higher disease activity, including more severe SSc-related complications [7,8,9,10]. Inflammation-based ratios have been less extensively studied in SSc. However, a meta-analysis from Zinellu et al. [11] revealed that patients with SSc had higher neutrophil-to-lymphocyte ratios (NLRs), platelet-to-lymphocyte ratios (PLRs), and monocyte-to-lymphocyte ratios (MLRs), which were also associated with ILD, PAH, and digital ulcers (DU). Nevertheless, their predictive value for SSc-specific complications remains to be clarified. Furthermore, clinical data on other ratios like the aspartate transaminase (AST)-to-alanine transaminase (ALT) ratio, ESR/CRP ratio, and CRP/albumin ratio, which have been analyzed in autoimmune diseases, are still lacking in SSc to the best of our knowledge [12,13,14].

The aim of this study was to investigate the associations of readily accessible inflammation-based ratios, SSc-specific clinical and serological parameters, and markers of endothelial dysfunction to assess the predictive role of these ratios for clinical outcome parameters and to evaluate potential longitudinal changes in these ratios during a three-year follow-up in patients with lcSSc.

## 2. Materials and Methods

### 2.1. Study Design and Patient Cohort

This study is a combined sub-analysis of two previously published studies including patients with lcSSc. The present analysis combines baseline and 3-year follow-up data from the same lcSSc cohort; 38 patients were included at baseline and 33 (86.8%) completed all follow-up visits, with 5 patients lost to follow-up. The same inclusion and exclusion criteria, sample handling, and laboratory protocols were applied at both time points. Thus, the current study does not represent an independent validation cohort, but a longitudinal analysis of a single cohort previously reported for endothelial function and vascular events [15,16]. The original baseline study investigated endothelial dysfunction in patients with lcSSc who were naïve to vasculopathy-mediated complications. It included 38 patients with confirmed lcSSc and 38 controls with diagnosed primary Raynaud’s phenomenon, matched 1:1 for age, sex, and race [15]. The respective follow-up study evaluated then the incidence of disease-specific complications and cardiovascular disease in lcSSc patients during a 3-year observational period [16]. The recent study included baseline assessments conducted between April 2019 and February 2020, while lcSSc patients were observed prospectively for three years until May 2023. Control subjects did not receive follow-up study visits due to the study design of the previous study [16]. For details on the study design, see Supplementary Figure S1, which provides a flow chart of the study process. The lcSSc cohort, laboratory protocols, and inclusion/exclusion criteria remained identical. Full details about the study designs have been previously described [15,16]. At baseline, measurements of inflammation-based ratios, endothelial dysfunction, and lcSSc-specific parameters were performed. Inflammation-based ratios and the development of lcSSc-specific disease complications, as well as cardiovascular disease, were recorded at annual study visits during the observational period.

Inclusion in the case group required a diagnosis of lcSSc based on the recent EULAR/ACR criteria [5]. Exclusion criteria at enrollment were age <18 years, presence of dcSSc or other connective tissue diseases, current or previous PAH, DU, endoscopically confirmed reflux, diabetes mellitus, and symptomatic atherosclerotic cardiovascular diseases. Additional exclusion criteria included recent pregnancy, malignancies, acute infections at enrollment, and intake of vasoactive medications (prostanoids, calcium channel blockers, phosphodiesterase-5 inhibitors, or endothelin receptor inhibitors) within 24 h prior to the study.

The primary endpoint of the current study was the difference in inflammation-based ratios between patients with lcSSc and controls with primary Raynaud’s phenomenon at baseline. Secondary endpoints included changes in inflammation-based ratios within the group of lcSSc patients between baseline and the last follow-up visit, associations between inflammation-based ratios, lcSSc-specific parameters, and parameters of endothelial dysfunction at baseline, as well as associations between inflammation-based ratios and the development of vascular and clinical events.

### 2.2. Biochemical Analyses

Blood samples were collected and parameters including neutrophils, lymphocytes, monocytes, platelets, mean platelet volume, ESR, CRP, AST, ALT, ferritin, fibrinogen, albumin, and high-density lipoprotein (HDL) were measured in routine laboratory work-up for the calculation of inflammation-based ratios. NLR, MLR, PLR, mean platelet volume-to-platelet ratio (MPVPR), mean platelet volume-to-lymphocyte ratio (MPVLR), ESR/CRP ratio, AST/ALT ratio, ferritin/ESR ratio, fibrinogen-to-albumin ratio (FAR), CRP/albumin ratio, monocyte/HDL ratio, lymphocyte/HDL ratio, and neutrophil/HDL ratio were calculated post hoc. Additionally, asymmetric dimethylarginine (ADMA), symmetric dimethylarginine (SDMA), homoarginine, arginine, CD31+/CD42b− endothelial microparticles (EMPs), and von Willebrand factor (vWF) antigen (vWF: Ag) and activity (vWF: Ac) were measured from blood samples at baseline. Parameters of the arginine metabolism were assessed using high-performance liquid chromatography [17,18]. EMPs were measured according to the recommendations published by Cossarizza et al. [19]. vWF: Ag and vWF Ac were measured in the same laboratory during routine laboratory work-up. Detailed information about the methods used for measuring the respective parameters of endothelial dysfunction have been previously published [15].

### 2.3. LcSSc-Specific Parameters

The capillaroscopic skin ulcer risk index (CSURI), microangiopathy evolution score (MES), modified Rodnan Skin Score (mRSS), UCLA SCTC GIT 2.0 total and constipation score, DETECT score, and EUSTAR index were defined as lcSSc-specific parameters. CSURI and MES were assessed using nailfold videocapillaroscopy of the second to fifth digits on both hands (Skinview, Optometron Ltd., Oskar-Messter str., Ismaning, Germany), while mRSS was recorded through physical examination [20,21,22]. UCLA SCTC GIT 2.0 total and constipation scores were assessed using standardized questionnaires, while the DETECT score and EUSTAR index were recorded according to the published data [23,24,25].

### 2.4. Vascular Assessment

Functional parameters of endothelial dysfunction, including flow-mediated dilation (FMD), nitroglycerine-mediated dilation (NMD), pulse wave velocity (PWV), and augmentation index (Aix) were assessed at baseline. Detailed methodological information about these parameters has been previously described [15]. In brief, FMD and NMD were measured according to the guidelines by Corretti et al. [26], while PWV and Aix were assessed with an oscillometric device (I.E.M. Mobil-O-Graph, I.E.M., Stolberg, Germany) using automated pulse wave analysis.

### 2.5. Vascular and Clinical Events

Microvascular events were defined as the development of new DU and PAH, while macrovascular events were defined as the development of new symptomatic coronary heart disease, carotid and vertebral artery disease, and peripheral artery disease during follow-up. Clinical events encompassed all micro- and macrovascular events, including additionally the development of new ILD, renal crisis, and esophageal dysfunction during follow-up. Further details about definitions and the recording of respective vascular and clinical events have been previously published [16].

### 2.6. Statistical Analysis

The Kolmogorov–Smirnov test was used to assess normal distribution. Continuous variables were expressed as means with standard deviations (SDs) if normally distributed or medians with interquartile ranges if non-normally distributed. Student’s *t*-test was applied for normally distributed data comparisons, and the Mann–Whitney U test was used for non-normally distributed data comparisons. The paired sample t-test was used to analyze differences in matched normally distributed samples, and the Wilcoxon signed-rank tests was used to analyze differences in matched non-normally distributed samples. Correlations were evaluated using Pearson’s coefficient for normally distributed variables and Spearman’s coefficient for non-normally distributed variables. In addition, a correlation heatmap illustrating the correlation coefficients between the main inflammation-based ratios, lcSSc-specific parameters, and key endothelial dysfunction parameters is provided in Supplementary Figure S2. For each outcome event, baseline inflammation-based ratios were compared between patients who developed the respective event during follow-up and those who did not. For this purpose, one-way ANOVA was used, which has only two levels. Each analysis for ANOVA compares only two groups (event yes/no), so classical post hoc procedures (Tukey, etc.) do not apply. Statistical analyses were adjusted for multiple testing using the Bonferroni–Holm correction due to the small sample size, given the exploratory and underpowered study design with a high number of tests, to reduce the risk of false positives at the expense of potentially increasing the risk of false negatives in this small cohort. The Bonferroni–Holm correction is used to control for multiple testing across markers/outcomes, not as a pairwise post hoc to distinguish more than two levels within a single ANOVA. Statistical significance was set at *p* < 0.05. All analyses were performed using SPSS version 29.0.

### 2.7. Ethics Statement

This research protocol received approval from the local ethics committee (protocol number EK 29-361 ex 16/17, approval date: 14 July 2017) and adhered to the principles outlined in the Helsinki Declaration of 1975, as revised in 2013. All subjects were thoroughly informed about the study details and provided their written informed consent.

## 3. Results

A total of 38 patients with diagnosed lcSSc and 38 controls with primary Raynaud’s phenomenon were included at the baseline of this study, while 33 patients with lcSSc (86.8%) completed all follow-up study visits and 5 patients with lcSSc (13.2%) were lost to follow-up during the three-year observational period. Patient characteristics and baseline data are provided in Supplementary Table S1, while Supplementary Table S2 presents the analysis of blood count, inflammatory and liver parameters, and HDL levels, comparing lcSSc patients and controls at baseline. Follow-up visits as well as the results of lcSSc-specific parameters, including the development of vascular and clinical events and the results of the parameters of endothelial dysfunction, have been previously published [15,16]. DU occurred in eight patients (24.2%), PAH in one patient (3.0%), ILD in two patients (6.1%), and symptomatic carotid and vertebral artery disease in one patient (3.0%).

Patients with lcSSc had significantly higher values for the NLR, MLR, FAR, monocyte/HDL ratio, and neutrophil/HDL ratio (all *p* < 0.05) compared to controls at baseline (Table 1). Furthermore, there was a trend for higher values of the PLR (*p* = 0.080), MPVLR (*p* = 0.063), and CRP/albumin ratio (*p* = 0.070), as well as for lower values of the ferritin/ESR ratio (*p* = 0.055) in lcSSc patients. Comparing inflammation-based ratios between baseline and last follow-up visit within lcSSc patients, the MLR, CRP/albumin ratio, monocyte/HDL ratio, and neutrophil/HDL ratio rose significantly from baseline to last follow-up visit after three years (all *p* < 0.05). The MPVPR and AST/ALT ratio revealed a statistically declining trend (*p* = 0.063; *p* = 0.052, respectively) between the baseline and last follow-up visit (Table 2).

### 3.1. Correlations of Inflammation-Based Ratios with lcSSc-Specific Parameters and Parameters of Endothelial Dysfunction

The Monocyte/HDL ratio correlated significantly negatively with the UCLA SCTC GIT total score (r = −0.469, *p* = 0.024) and significantly positively with the DETECT score step 2 (r = 0.453, *p* = 0.032). There was also a trend between the monocyte/HDL ratio and the DETECT score step 1 (r = 0.420, *p* = 0.072), as well as between the neutrophil/HDL ratio and the DETECT score step 1 (r = 0.425, *p* = 0.064), but without significance. Additionally, the CRP/albumin ratio and EUSTAR index revealed a significant positive correlation (r = 0.473, *p* = 0.024). The remaining correlation analysis of inflammation-based ratios and lcSSc-specific parameters is shown in Table 3.

In the correlation analysis between inflammation-based ratios and endothelial dysfunction, only a significant positive correlation between FAR and ADMA could be observed (r = 0.452, *p* = 0.044), without any further significant results or trends (Table 4).

The main correlation results of key inflammation-based ratios, lcSSc-specific parameters, and endothelial dysfunction parameters are depicted in Supplementary Figure S2 as a heatmap.

### 3.2. Associations of Inflammation-Based Ratios and Development of Vascular and Clinical Events in lcSSc

The development of PAH was significantly associated with the MLR and CRP/albumin ratio (*p* = 0.036, *p* = 0.006, respectively), and the development of ILD was significantly associated with the lymphocyte/HDL ratio (*p* = 0.004). No associations were found between inflammation-based ratios and DU nor between inflammation-based ratios and the composite end point parameter of vascular and clinical events (Table 5). Additional statistical information about the sum of squares, degree of freedom, and F-values of the ANOVA are listed in Supplementary Table S3.

## 4. Discussion

The recent study investigated the differences in several inflammation-based ratios and their associations with clinical and endothelial dysfunction parameters as well as with prospective outcome parameters in lcSSc patients. Comparing inflammation-based ratios between patients with lcSSc and controls, significant differences in the NLR, MLR, FAR, monocyte/HDL ratio, and neutrophil/HDL ratio were observed. Some of these findings are consistent with the existing literature, which described higher NLR and MLR values in patients with SSc [11]. Our recent study focused solely on lcSSc in contrast to the existing meta-analysis where the population was general SSc patients, whereby our findings extend the existing literature so that both ratios may serve as additional biomarkers for assessing inflammation in lcSSc without vasculopathy-mediated complications. The FAR, monocyte/HDL ratio, and neutrophil/HDL ratio were also significantly higher in lcSSc patients. These ratios have been predominantly studied in cardiovascular research. The FAR may reflect thromboinflammatory processes, while the monocyte/HDL ratio and neutrophil/HDL ratio combine the synergistic roles of inflammation and lipid metabolism. An elevation of those three ratios was also associated with major adverse cardiovascular events [27,28,29]. Regarding SSc, only the monocyte/HDL ratio has been previously investigated, showing higher levels in SSc patients, while data on the FAR and neutrophil/HDL ratio are currently lacking to the best of our knowledge [30]. Due to the limited data, it may be possible that some vasculopathic and inflammatory background processes, which promote clinical manifestations in SSc, may be better reflected by these ratios than by the single parameters, although larger studies are needed to prove this hypothesis. Other ratios such as the PLR and CRP/albumin ratio have been reported to be altered in autoimmune disorders, including systemic sclerosis [11,14]. However, in our study, these parameters did not show statistically significant differences between groups [11,14]. One explanation for this finding may be the small sample size. However, we also demonstrated that some of the elevated ratios at baseline changed significantly during the observational period, suggesting a dynamic effect of the vasculopathic and inflammatory background processes. Specifically, the MLR, CRP/albumin, monocyte/HDL, and neutrophil/HDL ratio showed significant changes during the follow-up period. These changes may be explained by dynamic shifts in systemic inflammation, immune cell activation, and vascular injury. The MLR changes reflect fluctuations in monocyte-driven inflammation and lymphocyte-mediated immune regulation. An elevated MLR is associated with increased disease activity and severity in SSc and other inflammatory conditions, likely due to monocyte recruitment and lymphopenia during active disease phases [31]. These ratios can vary over time as the inflammatory burden and immune response evolve. MPVPR is influenced by platelet activation and turnover, which increase systemic inflammation and vasculopathy. An increased MPVPR has been linked to more severe inflammatory states and worse outcomes in sepsis and chronic kidney disease, suggesting that platelet size and count are sensitive markers of ongoing inflammatory and thrombotic processes [32]. Changes in CRP/albumin over time indicate shifts in inflammatory activity and nutritional status and are associated with disease severity and mortality in acute and chronic illnesses [33]. The monocyte/HDL ratio reflects the interplay between pro-inflammatory leukocytes and anti-inflammatory, vasculoprotective HDL. These ratios are dynamic and may change with disease activity, lipid metabolism, and treatment [34].

The correlation analysis between inflammation-based ratios, lcSSc-specific parameters, and endothelial dysfunction revealed only occasional significant results. The monocyte/HDL ratio correlated negatively with the UCLA SCTC GIT total score and positively with the DETECT score step 2 points. The monocyte/HDL ratio is increasingly recognized as a marker of vascular inflammation and cardiovascular risk in SSc, since lower HDL and higher monocyte counts commonly reflect ongoing inflammation and endothelial dysfunction [35]. A significant negative correlation with the UCLA SCTC GIT total score suggests that a higher monocyte/HDL ratio is associated with less severe gastrointestinal involvement. This may reflect the pathophysiological mechanisms underlying gastrointestinal involvement in SSc, which are more related to microvascular damage, fibrosis, and gut dysbiosis than to systemic inflammation or atherogenesis [36]. However, the inverse correlation between the monocyte/HDL ratio and the UCLA SCTC GIT total score is counterintuitive, isolated, and requires confirmation in independent cohorts. Thus, while patients with a higher monocyte/HDL ratio might represent a vascular-inflammatory phenotype with less gastrointestinal damage, this finding should be interpreted cautiously. The significant correlation of the monocyte/HDL ratio with the DETECT score may reflect a pro-inflammatory, pro-atherogenic state that predisposes to PAH, although this ratio was not a predictor for PAH in our study [35,37]. The significant positive correlation between the CRP/albumin ratio and the EUSTAR index is mostly tautological, as CRP is a component of the EUSTAR index formula. This dependence limits the interpretation of the CRP/albumin ratio as an independent marker of disease activity.

The observed significant correlation between the FAR and ADMA may be rather coincidental due to multiple testing, but it could also reflect underlying pathophysiological interactions involving inflammation, endothelial dysfunction, and prothrombotic states. The FAR is increasingly recognized as a marker of systemic inflammation and prothrombotic risk, with an elevated FAR associated with adverse outcomes in cardiovascular disease, heart failure, and stroke [38,39]. The correlation between the FAR and ADMA may reflect shared inflammatory and vascular pathways rather than a direct biochemical interaction. This is consistent with the literature showing that both are independent predictors of poor cardiovascular outcomes, but their direct association is not robustly established in large cohorts [38,39,40,41,42]. Nevertheless, this correlation should be interpreted with caution due to the low statistical power of tests.

In our exploratory analysis, we also assessed whether baseline inflammation-based ratios were associated with the development of lcSSc complications during follow-up (Table 5). The MLR and the CRP/albumin ratio showed significant associations with incident PAH, and the lymphocyte/HDL ratio was associated with incident ILD, whereas no associations were observed for DU, vascular, or clinical events. Given the very small number of events, these findings should be interpreted rather as preliminary trends than robust predictors. In particular, the associations of the MLR and the CRP/albumin ratio with incident PAH, and of the lymphocyte/HDL ratio with incident ILD, are based on only one PAH case and two ILD cases in our cohort. Nevertheless, our findings are supported by recent evidence. A systematic review and meta-analysis demonstrated that the MLR is significantly elevated in SSc patients with PAH compared to those without complications, suggesting its utility as a non-invasive biomarker for PAH risk stratification [11]. Additionally, CRP elevation, and by extension the CRP/albumin ratio, is associated with an increased risk of progressive fibrosis and PAH in SSc, reflecting underlying inflammatory activity and poorer prognosis [43]. Regarding the lymphocyte/HDL ratio and its association with ILD, the current literature does not specifically validate this ratio as a biomarker for SSc-ILD. Instead, other hematological indices such as the NLR and PLR are more consistently associated with ILD in SSc, and monocyte/macrophage phenotypes have been linked to ILD severity and progression [11,43]. These results highlight the potential of simple inflammatory indices for pulmonary complication risk assessment in SSc, but, given the very small number of clinical events in our cohort, these associations must be interpreted cautiously and validated in larger prospective studies. Contrary to a previous study from Kim et al. [30], the monocyte/HDL ratio was not associated with the development of DU. Furthermore, no ratio was associated with the composite parameter of vascular or clinical events. Vascular and clinical events occurred only sporadically in lcSSc patients during the follow-up period, with newly developed DU in eight patients (24.2%), newly developed ILD in two patients (6.1%), newly developed PAH in one patient (3.0%), and newly developed transient ischemic attack in one patient (3.0%) [16]. While these association findings suggest that potential underlying vasculo-inflammatory pathways contributing to SSc-specific complications differ from each other, which can be quantified by selective inflammation-based ratios, they should be interpreted with caution.

Several limitations of this study should be acknowledged. Due to the exploratory design with a small sample size and very low event rates, our results are underpowered. Furthermore, as this analysis reuses the same lcSSc cohort from our previous publications without an independent validation sample, the generalizability is limited and the overfitting risk increased. The Bonferroni–Holm correction was applied to reduce false positives, but this also increases the risk of false negatives in underpowered analyses. Finally, the observational design precludes causal inferences regarding inflammation-based ratios and clinical outcomes.

## 5. Conclusions

In conclusion, this study provides new insights into the inflammatory landscape of lcSSc and demonstrates the utility of readily accessible inflammation-based ratios for assessing potential disease activity. Overall, our results should be considered hypothesis-generating. Given the small sample size, low event rates, and extensive multiple testing, the apparent associations between specific inflammation-based ratios and pulmonary complications should be interpreted cautiously and should be validated in larger prospective studies.

## Figures and Tables

**Table 1:** Bivariate analysis of inflammation-based ratios between patients with lcSSc and controls at baseline.

	LcSSc (n = 38)	Controls (n = 38)	*p*-Value
NLR, median (25–75th percentile)	2.40 (1.87–3.21)	2.00 (1.44–2.49)	**0.026**
MLR, mean (±SD)	0.28 ± 0.12	0.22 ± 0.08	**0.013**
PLR, mean (±SD)	181.58 ± 64.91	156.76 ± 55.60	0.080
MPVPR, median (25–75th percentile)	0.040 (0.029–0.051)	0.040 (0.030–0.050)	0.630
MPVLR, mean (±SD)	7.38 ± 2.60	6.47 ± 2.21	0.063
ESR/CRP ratio, median (25–75th percentile)	4.50 (2.40–10.31)	5.75 (2.77–11.49)	0.470
AST/ALT ratio, median (25–75th percentile)	1.19 (1.02–1.36)	1.23 (1.05–1.53)	0.436
Ferritin/ESR ratio, median (25–75th percentile)	7.00 (4.20–16.15)	9.77 (0.64–27.53)	0.055
FAR, mean (±SD)	69.89 ± 18.21	64.55 ± 12.20	**0.044**
CRP/albumin ratio, median (25–75th percentile)	0.23 (0.14–0.62)	0.18 (0.14–0.30)	0.070
Monocyte/HDL ratio, median (25–75th percentile)	0.20 (0.16–0.30)	0.16 (0.12–0.23)	**0.023**
Lymphocyte/HDL ratio, median (25–75th percentile)	0.80 (0.58–1.21)	0.80 (0.60–1.11)	0.975
Neutrophil/HDL ratio, median (25–75th percentile)	2.00 (1.43–2.83)	1.65 (1.15–1.94)	**0.035**

Abbreviations: ALT: alanine transaminase; AST: aspartate transaminase; CRP: C-reactive protein; ESR: erythrocyte sedimentation rate; FAR: fibrinogen-to-albumin ratio; HDL: high-density lipoprotein; MLR: monocyte-to-lymphocyte ratio; MPVPR: mean platelet volume-to-platelet ratio; MPVLR: mean platelet volume-to-lymphocyte ratio; NLR: neutrophil-to-lymphocyte ratio; PLR: platelet-to-lymphocyte ratio. Significant values are indicated in bold.

**Table 2:** Bivariate analysis of inflammation-based ratios between baseline and last follow-up visit within lcSSc patients.

	Baseline *	3-Year FUP	*p*-Value
NLR, median (25–75th percentile)	2.11 (1.80–3.23)	2.56 (1.75–3.36)	0.233
MLR, mean (±SD)	0.27 ± 0.12	0.34 ± 0.14	**<0.001**
PLR, mean (±SD)	175.05 ± 59.05	172.07 ± 59.21	0.668
MPVPR, median (25–75th percentile)	0.040 (0.030–0.049)	0.038 (0.029–0.051)	0.063
MPVLR, mean (±SD)	6.79 ± 2.24	6.41 ± 2.37	0.128
ESR/CRP ratio, median (25–75th percentile)	4.00 (2.11–10.00)	5.48 (2.66–10.50)	0.225
AST/ALT ratio, median (25–75th percentile)	1.19 (1.04–1.30)	1.00 (0.93–1.27)	0.052
Ferritin/ESR ratio, median (25–75th percentile)	7.00 (4.86–18.30)	10.17 (5.15–17.71)	0.689
FAR, mean (±SD)	67.53 ± 18.01	67.17 ± 14.63	0.679
CRP/albumin ratio, median (25–75th percentile)	0.20 (0.14–0.63)	0.24 (0.12–0.63)	**<0.001**
Monocyte/HDL ratio, median (25–75th percentile)	0.22 (0.17–0.32)	0.30 (0.21–0.36)	**<0.001**
Lymphocyte/HDL ratio, median (25–75th percentile)	0.81 (0.61–1.26)	0.88 (0.70–1.13)	0.360
Neutrophil/HDL ratio, median (25–75th percentile)	2.02 (1.53–2.84)	2.38 (1.64–3.37)	**0.045**

Abbreviations: ALT: alanine transaminase; AST: aspartate transaminase; CRP: C-reactive protein; ESR: erythrocyte sedimentation rate; FAR: fibrinogen-to-albumin ratio; FUP: follow-up; HDL: high-density lipoprotein; MLR: monocyte-to-lymphocyte ratio; MPVPR: mean platelet volume-to-platelet ratio; MPVLR: mean platelet volume-to-lymphocyte ratio; NLR: neutrophil-to-lymphocyte ratio; PLR: platelet-to-lymphocyte ratio. *: Five patients were excluded from the baseline due to lost to follow-up during observation period. Significant values are indicated in bold.

**Table 3:** Correlation analysis of inflammation-based ratios and clinical parameters within patients with lcSSc at baseline visit adjusted for multiple testing.

		NLR	MLR	PLR	MPVPR	MPVLR	ESR/CRP Ratio	AST/ALT Ratio	Ferritin/ESR Ratio	FAR	CRP/Albumin Ratio	Monocyte/HDL Ratio	Lymphocyte/HDL Ratio	Neutrophi/HDL Ratio
**CSURI**	r	0.151	0.088	−0.069	−0.183	−0.234	0.175	0.208	0.034	0.292	0.096	0.295	0.155	0.272
	*p*	>0.999	>0.999	>0.999	>0.999	>0.999	>0.999	>0.999	>0.999	0.600	>0.999	0.576	>0.999	0.264
**MES**	r	0.082	0.075	−0.080	−0.092	−0.057	0.191	−0.055	−0.017	0.084	−0.036	0.088	0.056	0.044
	*p*	>0.999	>0.999	>0.999	>0.999	>0.999	>0.999	>0.999	>0.999	>0.999	>0.999	>0.999	>0.999	>0.999
**mRSS**	r	0.098	−0.080	0.048	−0.032	−0.011	−0.187	0.232	0.252	0.001	0.347	−0.099	0.057	0.063
	*p*	>0.999	>0.999	>0.999	>0.999	>0.999	>0.999	>0.999	>0.999	>0.999	0.264	>0.999	>0.999	>0.999
**UCLA SCTC GIT total score**	r	−0.124	−0.271	0.128	−0.014	0.108	−0.144	−0.006	0.191	−0.192	0.072	**−0.469**	−0.132	−0.262
	*p*	>0.999	0.800	>0.999	>0.999	>0.999	>0.999	>0.999	>0.999	>0.999	>0.999	**0.024**	>0.999	0.896
**UCLA SCTC GIT constipation score**	r	−0.085	−0.062	0.163	0.026	0.131	−0.157	−0.146	−0.063	−0.158	−0.035	−0.067	−0.045	−0.109
	*p*	>0.999	>0.999	>0.999	>0.999	>0.999	>0.999	>0.999	>0.999	>0.999	>0.999	>0.999	>0.999	>0.999
**DETECT score step 1**	r	0.014	0.111	−0.182	0.002	−0.131	−0.275	0.159	−0.134	0.064	0.336	0.420	0.045	0.425
	*p*	>0.999	>0.999	>0.999	>0.999	>0.999	0.752	>0.999	>0.999	>0.999	0.312	0.072	>0.999	0.064
**DETECT score step 2**	r	−0.029	0.109	−0.211	0.020	−0.142	−0.233	0.110	−0.150	0.021	0.261	**0.453**	−0.096	0.388
	*p*	>0.999	>0.999	>0.999	>0.999	>0.999	>0.999	>0.999	>0.999	>0.999	0.904	**0.032**	>0.999	0.128
**EUSTAR index**	r	0.165	−0.070	0.023	−0.170	−0.177	−0.395	0.273	0.158	0.040	**0.473**	0.091	0.179	0.248
	*p*	>0.999	>0.999	>0.999	>0.999	>0.999	0.112	0.776	>0.999	>0.999	**0.024**	>0.999	>0.999	>0.999

Abbreviations: ALT: alanine transaminase; AST: aspartate transaminase; CRP: C-reactive protein; CSURI: capillaroscopic skin ulcer risk index; eGFR: estimated glomerular filtration rate; ESR: erythrocyte sedimentation rate; FAR: fibrinogen-to-albumin ratio; FUP: follow-up; HDL: high-density lipoprotein; MES: microangiopathy evolution score; MLR: monocyte-to-lymphocyte ratio; MPVPR: mean platelet volume-to-platelet ratio; MPVLR: mean platelet volume-to-lymphocyte ratio; mRSS: modified Rodnan Skin Score; NLR: neutrophil-to-lymphocyte ratio; PLR: platelet-to-lymphocyte ratio. Significant values are indicated in bold.

**Table 4:** Correlation matrix of inflammation-based ratios and parameters of endothelial dysfunction within patients with lcSSc adjusted for multiple testing.

		NLR	MLR	PLR	MPVPR	MPVLR	ESR/CRP Ratio	AST/ALT Ratio	Ferritin/ESR Ratio	FAR	CRP/Albumin Ratio	Monocyte/HDL Ratio	Lymphocyte/HDL Ratio	Neutrophil/HDL Ratio
**FMD**	r	−0.444	−0.234	−0.101	0.017	−0.175	0.012	0.001	0.148	−0.018	−0.107	0.076	0.266	−0.151
	*p*	0.055	>0.999	>0.999	>0.999	>0.999	>0.999	>0.999	>0.999	>0.999	>0.999	>0.999	>0.999	>0.999
**NMD**	r	−0.219	−0.229	−0.131	−0.155	−0.177	0.339	0.084	0.183	−0.173	−0.448	−0.007	0.162	−0.004
	*p*	>0.999	>0.999	>0.999	>0.999	>0.999	0.594	>0.999	>0.999	>0.999	0.099	>0.999	>0.999	>0.999
**PWV**	r	0.225	0.384	0.036	−0.068	−0.040	0.220	0.137	−0.335	0.340	0.203	0.165	−0.168	0.112
	*p*	>0.999	0.187	>0.999	>0.999	>0.999	>0.999	>0.999	0.427	0.407	>0.999	>0.999	>0.999	>0.999
**Aix**	r	−0.175	0.045	−0.152	0.077	−0.273	0.331	−0.054	−0.090	0.244	−0.048	0.101	0.208	0.019
	*p*	>0.999	>0.999	>0.999	>0.999	>0.999	0.473	>0.999	>0.999	>0.999	>0.999	>0.999	>0.999	>0.999
**CD31+/CD42b− EMP**	r	−0.124	−0.072	−0.119	−0.084	−0.154	0.132	−0.107	−0.164	0.239	−0.030	0.182	0.141	−0.012
	*p*	>0.999	>0.999	>0.999	>0.999	>0.999	>0.999	>0.999	>0.999	>0.999	>0.999	>0.999	>0.999	>0.999
**ADMA**	r	0.032	0.020	0.200	−0.404	−0.029	−0.002	−0.014	−0.277	**0.452**	0.194	0.017	−0.050	0.065
	*p*	>0.999	>0.999	>0.999	0.132	>0.999	>0.999	>0.999	>0.999	**0.044**	>0.999	>0.999	>0.999	>0.999
**SDMA**	r	0.018	0.212	0.241	−0.212	0.066	0.078	0.096	−0.326	0.252	−0.008	0.151	−0.136	−0.025
	*p*	>0.999	>0.999	>0.999	>0.999	>0.999	>0.999	>0.999	0.506	>0.999	>0.999	>0.999	>0.999	>0.999
**Arginine**		0.146	0.165	0.246	−0.370	0.065	−0.061	0.195	−0.278	0.152	0.125	0.003	−0.098	0.039
		>0.999	>0.999	>0.999	0.242	>0.999	>0.999	>0.999	>0.999	>0.999	>0.999	>0.999	>0.999	>0.999
**Homoarginine**		0.266	0.142	0.119	0.127	0.166	−0.117	−0.224	0.154	−0.115	−0.125	0.053	−0.011	0.140
		>0.999	>0.999	>0.999	>0.999	>0.999	>0.999	>0.999	>0.999	>0.999	>0.999	>0.999	>0.999	>0.999
**vWF:Ac**		0.317	0.11	−0.062	0.109	0.110	0.134	0.076	−0.256	0.183	0.025	0.068	−0.075	0.193
		0.616	>0.999	>0.999	>0.999	>0.999	>0.999	>0.999	>0.999	>0.999	>0.999	>0.999	>0.999	>0.999
**vWF:Ag**	r	0.338	0.062	0.011	−0.076	0.072	0.124	0.149	−0.234	0.233	0.116	−0.048	−0.126	0.182
	*p*	0.451	>0.999	>0.999	>0.999	>0.999	>0.999	>0.999	>0.999	>0.999	>0.999	>0.999	>0.999	>0.999

Abbreviations: ADMA: asymmetric dimethylarginine; Aix: augmentation index; ALT: alanine transaminase; AST: aspartate transaminase; CRP: C-reactive protein; EMP: endothelial-derived microparticles; ESR: erythrocyte sedimentation rate; FAR: fibrinogen-to-albumin ratio; FMD: flow-mediated dilation; HDL: high-density lipoprotein; MLR: monocyte-to-lymphocyte ratio; MPVPR: mean platelet volume-to-platelet ratio; MPVLR: mean platelet volume-to-lymphocyte ratio; NLR: neutrophil-to-lymphocyte ratio; NMD: nitroglycerin-mediated dilation; PLR: platelet-to-lymphocyte ratio; PWV: pulse wave velocity; SDMA: symmetric dimethylarginine. vWF:Ac: von Willebrand factor activity; vWF:Ag: von Willebrand factor antigen. Significant values are indicated in bold.

**Table 5:** One-way ANOVA between development of vascular and clinical events during follow-up * and baseline inflammation-based ratios in patients with lcSSc adjusted with multiple testing. Numbers are given as *p*-values from the ANOVA.

	DU	PAH	Microvascular Event	Macrovascular Event	ILD	Clinical Event
**NLR**	>0.999	0.180	>0.999	>0.999	>0.999	>0.999
**MLR**	>0.999	**0.036**	>0.999	>0.999	>0.999	>0.999
**PLR**	>0.999	0.312	>0.999	>0.999	0.402	>0.999
**MPVPR**	>0.999	>0.999	>0.999	>0.999	>0.999	>0.999
**MPVLR**	>0.999	>0.999	>0.999	>0.999	0.612	0.714
**ESR/CRP ratio**	>0.999	>0.999	>0.999	>0.999	0.684	>0.999
**AST/ALT ratio**	>0.999	>0.999	>0.999	>0.999	>0.999	0.768
**Ferritin/ESR ratio**	>0.999	>0.999	>0.999	>0.999	>0.999	>0.999
**FAR**	>0.999	0.120	>0.999	>0.999	>0.999	>0.999
**CRP/albumin ratio**	>0.999	**0.006**	0.270	>0.999	>0.999	0.414
**Monocyte/HDL ratio**	>0.999	0.186	>0.999	>0.999	0.792	>0.999
**Lymphocyte/HDL ratio**	>0.999	>0.999	>0.999	>0.999	**0.004**	0.864
**Neutrophil/HDL ratio**	>0.999	0.666	>0.999	0.864	>0.999	0.864

Abbreviations: ALT: alanine transaminase; AST: aspartate transaminase; CRP: C-reactive protein; DUs: digital ulcers; ESR: erythrocyte sedimentation rate; FAR: fibrinogen-to-albumin ratio; HDL: high-density lipoprotein; ILD: interstitial lung disease; MLR: monocyte-to-lymphocyte ratio; MPVPR: mean platelet volume-to-platelet ratio; MPVLR: mean platelet volume-to-lymphocyte ratio; NLR: neutrophil-to-lymphocyte ratio; PAH: pulmonary arterial hypertension; PLR: platelet-to-lymphocyte ratio. *: Event groups (with vs. without event) during follow-up: PAH: 1 vs. 32 patients; ILD: 2 vs. 31 patients; DU: 8 vs. 25 patients; microvascular event: 9 vs. 24 patients; macrovascular event: 1 vs. 32 patients; clinical event: 10 vs. 23 patients. Significant values are indicated in bold.

## Data Availability

The data that support the findings of this study are available from the corresponding author upon request.

## Supplementary Materials

The supporting information can be downloaded at https://www.mdpi.com/article/10.3390/jcm14248806/s1, Figure S1: Flow diagram of the study design; Figure S2: Heatmap of correlation correlations between main inflammation-based ratios, lcSSc-specific parameters, and endothelial dysfunction markers in lcSSc patients at baseline. Significant correlations after Bonferroni-Holm correction are marked with * (*p* < 0.05); Table S1: Patients’ characteristics at study inclusion; Table S2: Bivariate analysis of blood count, inflammatory and liver parameters as well as HDL between patients with lcSSc and controls at baseline; Table S3: Sum of squares, degree of freedom and F-values of the one-way ANOVA between development of vascular and clinical events during follow-up and baseline inflammation-based ratios in patients with lcSSc.

## Abbreviations

The following abbreviations are used in this manuscript:

ACR	American College of Rheumatology
ADMA	Asymmetric dimethylarginine
Aix	Augmentation index
CSURI	Capillaroscopic skin ulcer index
CRP	C-reactive protein
dcSSc	Diffuse cutaneous systemic sclerosis
DU	Digital ulcers
EMP	Endothelial microparticles
ESR	Erythrocyte sedimentation rate
EULAR	European League Against Rheumatism
EUSTAR	European Scleroderma Trials and Research Group
FAR	Fibrinogen-to-albumin ratio
FMD	Flow-mediated dilation
ILD	Interstitial lung disease
lcSSc	Limited cutaneous systemic sclerosis
MLR	Monocyte-to-lymphocyte ratio
MPVLR	Mean platelet volume-to-lymphocyte ratio
MPVPR	Mean platelet volume-to-platelet ratio
mRSS	Modified Rodnan Skin Score
NLR	Neutrophil-to-lymphocyte ratio
NMD	Nitroglycerin-mediated dilatation
NVC	Nail fold videocapillaroscopy
PAH	Pulmonary arterial hypertension
PLR	Platelet-to-lymphocyte ratio
PWV	Pulse wave velocity
SD	Standard deviations
SDMA	Symmetric dimethylarginine
SSc	Systemic sclerosis
vWF	von Willebrand factor
