# Supplementary material for: Association of Inflammation-Based Ratios with Endothelial Dysfunction Markers and Clinical Parameters in Limited Cutaneous Systemic Sclerosis
Source: J Clin Med. 2025 Dec 12;14(24):8806. doi: 10.3390/jcm14248806 (PMC12733724; doi:10.3390/jcm14248806)
Supplement: Supplementary file 1 [file jcm-14-08806-s001.zip › jcm-3962661-supplementary.pdf]

Supplementary Table S1. Patients' characteristics at study inclusion.

	**LcSSc**	**Controls**	**p-value**
Number of patients, n (%)	38 (50)	38 (50)	>0.999
Disease duration, mean (± SD)	7.1 ± 5.8	5.7 ± 3.2	0.191
Sex, n (%)			
Male	2 (5.3)	2 (5.3)	>0.999
Female	36 (94.7)	36 (94.7)	>0.999
Age (years), mean (± SD)	57.9 ± 9.2	57.2 ± 9.0	0.622
BMI (kg/m2), mean (± SD)	23.61 ± 3.46	22.86 ± 3.53	0.487
Smoking, n (%)			
Active smokers	4 (10.5)	7 (18.4)	0.516
Ex-smokers	8 (21.1)	8 (21.1)	>0.999
Arterial hypertension, n (%)	14 (36.8)	12 (31.6)	0.809
Hyperlipidemia, n (%)	20 (52.6)	12 (31.6)	0.103
HbA1c (mmol/mol), median (25-75th percentile)	35 (34-37)	37 (34-38)	0.308
Malignancies, n (%)			
Recent	0 (0)	0 (0)	>0.999
Prior	4 (10.5)	1 (2.6)	0.358
Prior interstitial lung disease, n (%)	2 (5.3)	0 (0)	0.493
Prior esophageal involvement, n (%)	9 (23.7)	0 (0)	0.002
Prior renal involvement, n (%)	2 (5.3)	0 (0)	0.493
Medication, n (%)			
ACE inhibitors/ARB	7 (18.4)	6 (15.8)	>0.999
Beta blockers	3 (7.9)	4 (10.5)	>0.999
Calcium channel blockers	6 (15.8)	5 (13.2)	>0.999
Diuretics	2 (5.3)	3 (7.9)	>0.999
Platelet aggregation inhibitors	6 (15.8)	4 (10.5)	0.736
Anticoagulants	3 (7.9)	0 (0)	0.240
Statins	3 (7.9)	3 (7.9)	>0.999
Immunosuppression, n (%)			
Cortisone	3 (7.9)	0 (0)	0.240
Methotrexate	1 (2.6)	0 (0)	>0.999
Mycophenolate mofetil	2 (5.3)	0 (0)	0.493
Rituximab	1 (2.6)	0 (0)	>0.999
Hydroxychloroquine	2 (5.3)	0 (0)	0.493
Abatacept	1 (2.6)	0 (0)	>0.999

Abbreviations: ACE: angiotensin converting enzyme; ARB: Angiotensin II receptor blockers; BMI: body-mass-index; PAH: pulmonary arterial hypertension.

Supplementary Table S2. Bivariate analysis of blood count, inflammatory and liver parameters as well as HDL between patients with lcSSc and controls at baseline.

	**LcSSc**	**Controls**	**p-value**
White blood cells (10^9/L), median (25th-75th percentile)	5.72 (4.95-6.49)	5.58 (4.50-6.50)	0.451
Neutrophils	3.4 (3.0-4.4)	3.3 (2.7-3.9)	0.316
Lymphocytes	1.5 (1.2-1.8)	1.7 (1.4-2.2)	**0.049**
Monocytes	0.4 (0.3-0.5)	0.4 (0.3-0.4)	0.348
Platelets (10^9/L), mean (± SD)	260 (60)	258 (62)	0.903
MPV (fl), mean (± SD)	10.4 (1.0)	10.5 (0.6)	0.906
ESR (mm/h), median (25th-75th percentile)	9 (4-13)	7 (3-12)	0.132
CRP (mg/L), median (25th-75th percentile)	1.0 (0.6-2.9)	0.8 (0.6-1.4)	0.108
Ferritin (ng/ml), median (25th-75th percentile)	76 (36-94)	83 (37-207)	0.228
AST (U/L), median (25th-75th percentile)	25 (21-29)	23 (20-27)	0.323
ALT (U/L), median (25th-75th percentile)	21 (16-28)	20 (15-25)	0.526
Fibrinogen (mg/dl), mean (± SD)	330 (57)	303 (55)	**0.046**
Albumin (g/dl), mean (± SD)	4.62 (0.27)	4.71 (0.28)	0.139
HDL (mg/dl), mean (± SD)	72.5 (17.2)	81.7 (19.5)	**0.032**

Abbreviations: ALT: alanine transaminase; AST: aspartate transaminase; CRP: C-reactive-protein; ESR: erythrocyte sedimentation rate; HDL: high-density lipoprotein; MPV: mean platelet volume

Supplemental Table S3. Sum of squares, degree of freedom and F-values of the one-way ANOVA between development of vascular and clinical events during follow-up and baseline inflammation-based ratios in patients with lcSSc.

		DU			PAH		
		Sum of squares	df	F	Sum of squares	df	F
**NLR**	Between groups	2.437	1	1.463	9.048	1	6.232
	Within groups	51.620	31		45.009	31	
	Total	54.057	32		54.057	32	
**MLR**	Between groups	0.006	1	0.406	0.096	1	8.591
	Within groups	0.438	31		0.347	31	
	Total	0.443	32		0.443	32	
**PLR**	Between groups	473.149	1	0.132	13039.878	1	4.102
	Within groups	111119.019	31		98552.290	31	
	Total	111592.168	32		111592.168	32	
**MPVPR**	Between groups	0.000	1	0.313	0.000	1	1.021
	Within groups	0.005	31		0.005	31	
	Total	0.005	32		0.005	32	
**MPVLR**	Between groups	4.437	1	0.882	0.569	1	0.110
	Within groups	155.900	31		159.768	31	
	Total	160.337	32		160.337	32	
**ESR/CRP ratio**	Between groups	2.308	1	0.063	38.373	1	1.080
	Within groups	1137.703	31		1101.638	31	
	Total	1140.011	32		1140.011	32	
**AST/ALT ratio**	Between groups	0.248	1	1.301	0.001	1	0.003
	Within groups	5.914	31		6.162	31	
	Total	6.163	32		6.163	32	
**Ferritin/ESR ratio**	Between groups	82.220	1	0.056	281.818	1	0.193
	Within groups	45528.081	31		45328.483	31	
	Total	45610.301	32		45610.301	32	
**FAR**	Between groups	74.444	1	0.224	1693.483	1	6.043
	Within groups	10305.778	31		8686.739	31	
	Total	10380.222	32		10380.222	32	
**CRP/albumin ratio**	Between groups	0.029	1	0.008	102.295	1	249.253
	Within groups	144.989	31		12.723	31	
	Total	115.018	32		115.018	32	
**Monocytes/HDL ratio**	Between groups	0.012	1	1.633	0.033	1	5.127
	Within groups	0.219	31		0.198	31	
	Total	0.231	32		0.231	32	
**Lymphocytes/HDL ratio**	Between groups	0.001	1	0.003	0.058	1	0.315
	Within groups	5.744	31		5.687	31	
	Total	5.745	32		5.745	32	
**Neutrophils/HDL ratio**	Between groups	1.348	1	1.189	2.916	1	2.693
	Within groups	35.135	31		33.566	31	
	Total	36.482	32		36.482	32	
		**Microvascular event**			**Macrovascular event**		
		Sum of squares	df	F	Sum of squares	df	F
**NLR**	Between groups	0.371	1	0.214	0.546	1	0.317
	Within groups	53.686	31		53.511	31	

	Total	54.057	32		54.057	32	
**MLR**	Between groups	0.001	1	0.042	0.003	1	0.216
	Within groups	0.443	31		0.440	31	
	Total	0.443	32		0.443	32	
**PLR**	Between groups	5.283	1	0.001	2878.360	1	0.821
	Within groups	111586.885	31		108713.808	31	
	Total	111592.168	32		111592.168	32	
**MPVPR**	Between groups	0.000	1	0.427	0.000	1	0.423
	Within groups	0.005	31		0.005	31	
	Total	0.005	32		0.005	32	
**MPVLR**	Between groups	3.835	1	0.760	0.545	1	0.106
	Within groups	156.503	31		159.792	31	
	Total	160.337	32		160.337	32	
**ESR/CRP ratio**	Between groups	11.136	1	0.306	1.094	1	0.030
	Within groups	1128.875	31		1138.917	31	
	Total	1140.011	32		1140.011	32	
**AST/ALT ratio**	Between groups	0.325	1	1.727	0.067	1	0.340
	Within groups	5.837	31		6.069	31	
	Total	6.163	32		6.163	32	
**Ferritin/ESR ratio**	Between groups	393.932	1	0.270	189.186	1	0.129
	Within groups	45216.369	31		45421.115	31	
	Total	45610.301	32		45610.301	32	
**FAR**	Between groups	48.293	1	0.145	0.904	1	0.003
	Within groups	10331.929	31		10379.318	31	
	Total	10380.222	32		10380.222	32	
**CRP/albumin ratio**	Between groups	14.196	1	4.362	0.191	1	0.052
	Within groups	100.822	31		114.827	31	
	Total	115.018	32		115.018	32	
**Monocytes/HDL ratio**	Between groups	0.000	1	0.009	0.012	1	1.711
	Within groups	0.231	31		0.219	31	
	Total	0.231	32		0.231	32	
**Lymphocytes/HDL ratio**	Between groups	0.022	1	0.120	0.339	1	1.944
	Within groups	5.723	31		5.406	31	
	Total	5.745	32		5.745	32	
**Neutrophils/HDL ratio**	Between groups	0.044	1	0.038	0.398	1	0.342
	Within groups	36.438	31		36.084	31	
	Total	36.482	32		36.482	32	
		**ILD**			**Clinical events**		
		Sum of squares	df	F	Sum of squares	df	F
**NLR**	Between groups	2.293	1	1.373	1.424	1	0.406
	Within groups	51.764	31		52.633	31	
	Total	54.057	32		54.057	32	
**MLR**	Between groups	0.019	1	1.390	0.003	1	0.110
	Within groups	0.424	31		0.440	31	
	Total	0.443	32		0.443	32	
**PLR**	Between groups	11597.599	1	3.595	3277.619	1	0.454
	Within groups	99994.569	31		108314.549	31	
	Total	111592.168	32		111592.168	32	

**MPVPR**	Between groups	0.000	1	0.063	0.000	1	0.948
	Within groups	0.005	31		0.004	31	
	Total	0.005	32		0.005	32	
**MPVLR**	Between groups	13.488	1	2.847	12.266	1	1.243
	Within groups	146.850	31		148.071	31	
	Total	160.337	32		160.337	32	
**ESR/CRP ratio**	Between groups	89.675	1	2.647	1.954	1	0.026
	Within groups	1050.336	31		1138.057	31	
	Total	1140.011	32		1140.011	32	
**AST/ALT ratio**	Between groups	0.172	1	0.892	0.460	1	1.211
	Within groups	5.990	31		5.702	31	
	Total	6.163	32		6.163	32	
**Ferritin/ESR ratio**	Between groups	450.381	1	0.309	689.200	1	0.230
	Within groups	45159.920	31		44921.101	31	
	Total	45610.301	32		45610.301	32	
**FAR**	Between groups	287.651	1	0.884	272.027	1	0.404
	Within groups	10092.571	31		10108.194	31	
	Total	10380.222	32		10380.222	32	
**CRP/albumin ratio**	Between groups	0.622	1	0.168	13.575	1	2.007
	Within groups	114.397	31		101.443	31	
	Total	115.018	32		115.018	32	
**Monocytes/HDL ratio**	Between groups	0.017	1	2.397	0.012	1	0.831
	Within groups	0.214	31		0.219	31	
	Total	0.231	32		0.231	32	
**Lymphocytes/HDL ratio**	Between groups	1.855	1	14.779	0.582	1	1.691
	Within groups	3.890	31		5.163	31	
	Total	5.745	32		5.745	32	
**Neutrophils/HDL ratio**	Between groups	0.783	1	0.680	0.421	1	0.175
	Within groups	35.699	31		36.062	31	
	Total	36.482	32		36.482	32	

Abbreviations: ALT: alanine transaminase; AST: aspartate transaminase; CRP: C-reactive-protein; df: degree of freedom; DU: digital ulcers; ESR: erythrocyte sedimentation rate; FAR: fibrinogen-to-albumin ratio; HDL: high-density lipoprotein; ILD: interstitial lung disease; MLR: monocyte-to-lymphocyte ratio; MPVPR: mean-platelet-volume-to-platelet ratio; MPVLR: mean-platelet-volume-to-lymphocyte ratio; NLR: neutrophil-to-lymphocyte ratio; PAH: pulmonary arterial hypertension; PLR: platelet-to-lymphocytes ratio

Supplementary Figure S1. Flow diagram of the study design.

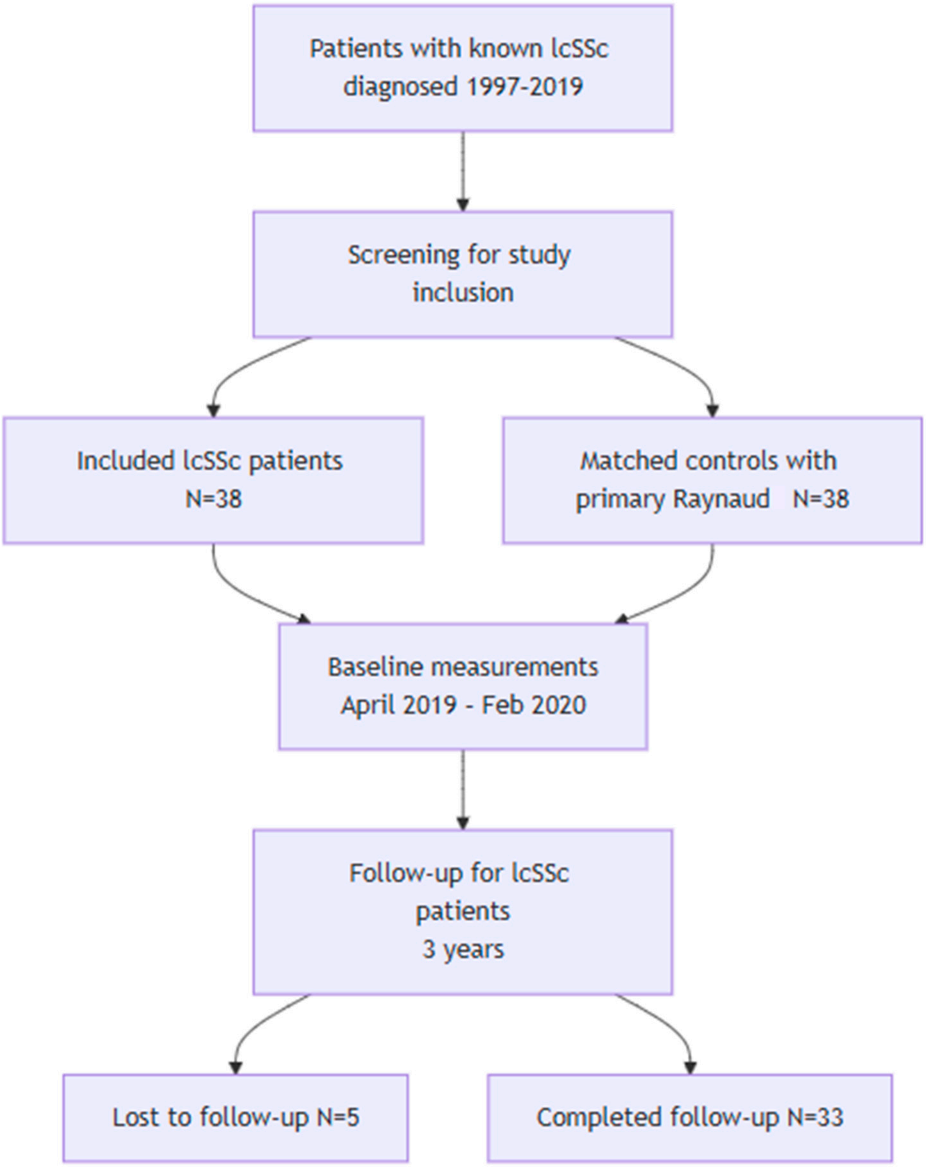

Supplementary Figure S2. Heatmap of correlation correlations between main inflammation-based ratios, lcSSc-specific parameters, and endothelial dysfunction markers in lcSSc patients at baseline. Significant correlations after Bonferroni-Holm correction are marked with * (p<0.05).

	**NLR**	**MLR**	**FAR**	**CRP/albumin ratio**	**Monocytes/HDL ratio**	**Lymphocytes/HDL ratio**	**Neutrophils/HDL ratio**	**UCLA SCTC GIT Score**	**DETECT score step 2**	**EUSTAR index**	**ADMA**
**NLR**	1.00	0.15	0.29	0.10	0.30	0.10	0.27	-0.12	-0.03	0.17	0.03
**MLR**	0.15	1.00	0.35	0.34	0.45	-0.10	0.39	-0.27	0.11	-0.07	0.02
**FAR**	0.29	0.35	1.00	0.20	0.26	0.09	0.18	-0.19	0.02	0.04	**0.45***
**CRP/albumin ratio**	0.10	0.34	0.20	1.00	0.07	0.07	0.06	-0.19	0.26	**0.47***	0.19
**Monocytes/HDL ratio**	0.30	0.45	0.26	0.07	1.00	-0.13	0.39	**-0.47***	**0.45***	0.09	0.02
**Lymphocytes/HDL ratio**	0.10	-0.10	0.09	0.07	-0.13	1.00	0.09	-0.13	-0.10	0.09	-0.05
**Neutrophils/HDL ratio**	0.27	0.39	0.18	0.06	0.39	0.09	1.00	-0.26	0.39	0.18	0.07
**UCLA SCTC GIT Score**	-0.12	-0.27	-0.19	-0.19	**-0.47***	-0.13	-0.26	1.00	-0.23	-0.40	0.02
**DETECT score step 2**	-0.03	0.11	0.02	0.26	**0.45***	-0.10	0.39	-0.23	1.00	0.11	-0.15
**EUSTAR index**	0.17	-0.07	0.04	**0.47***	0.09	0.09	0.18	-0.40	0.11	1.00	0.02
**ADMA**	0.03	0.02	**0.45***	0.19	0.02	-0.05	0.07	0.02	-0.15	0.02	1.00

Abbreviations: ADMA: asymmetric dimethylarginine; Aix: CRP: C-reactive-protein; FAR: fibrinogen-to-albumin ratio; HDL: high-density lipoprotein; MLR: monocyte-to-lymphocyte ratio; NLR: neutrophil-to-lymphocyte ratio.
